# Social Frailty Is Associated With the Risk of Malnutrition Among Community-Dwelling Older Adults

**DOI:** 10.1093/geroni/igaa057.1281

**Published:** 2020-12-16

**Authors:** Seoyoon Jane Lee, Miji Kim, Hayoung Shim, Heeeun Jung, Yuri Seo, Chang Won Won, Jane Lee

**Affiliations:** 1 Elderly Frailty Research Center, Kyung Hee University, Seoul, Republic of Korea; 2 College of Medicine, Kyung Hee University, Seoul, Republic of Korea; 3 Kyung Hee University, Seoul, Korea, Seoul, Republic of Korea; 4 Graduate School, Kyung Hee University, Seoul, Republic of Korea; 5 Kyung Hee University Medical Center, Seoul, Korea, Seoul, Republic of Korea

## Abstract

Social frailty does not merely affect the level of socialization, but also the means of obtaining resources for the daily lives of older adults. It is highly associated with the quality of life during advanced age. In this study, we analyzed the association between the Mini-Nutritional Assessment (MNA®) score (range 0-30) and 18 singular items and social frailty status of community-dwelling older adults. A total of 2,552 community-dwelling older adults aged 70-84 years (mean age 76.9±4.0, 51.8% female) from the Korean Frailty and Aging Cohort Study (KFACS) were assessed. The social frailty status was assessed in four categories- absence of economic resources, social resources, social activities, and social interactions. A higher MNA® score indicates better nutritional status. The prevalence of social frailty in older adults was 27.9% (mean age 78.1±4.0, 67.7% female). Approximately 40% of the participants were at risk of malnutrition or malnourished (p<0.001), while socially robust group accounted for 23% (p<0.001). They were at a higher risk of a lower MNA® score (odds ratio [OR] 0.90, 95% confidence interval [CI], 0.84-0.96). Socially-frail older adults have a higher possibility of not eating three full meals a day (OR 2.33, 95% CI 1.19-4.55) which increases the risk of malnutrition. In conclusion, social frailty, as a means of linking resources -including economic and social capitals- to the older adult population directly impacts the risk of malnutrition and requires an appropriate intervention.

